# Preliminary Evidence for the Amplification of Global Warming in Shallow, Intertidal Estuarine Waters

**DOI:** 10.1371/journal.pone.0141529

**Published:** 2015-10-28

**Authors:** Autumn Oczkowski, Richard McKinney, Suzanne Ayvazian, Alana Hanson, Cathleen Wigand, Erin Markham

**Affiliations:** 1 Atlantic Ecology Division, United States Environmental Protection Agency, Narragansett, Rhode Island, United States of America; 2 Graduate School of Oceanography, University of Rhode Island, Narragansett, Rhode Island, United States of America; CSIR- National institute of oceanography, INDIA

## Abstract

Over the past 50 years, mean annual water temperature in northeastern U.S. estuaries has increased by approximately 1.2°C, with most of the warming recorded in the winter and early spring. A recent survey and synthesis of data from four locations in Southern Rhode Island has led us to hypothesize that this warming may be amplified in the shallow (<1 m), nearshore portions of these estuaries. While intertidal areas are not typically selected as locations for long-term monitoring, we compiled data from published literature, theses, and reports that suggest that enhanced warming may be occurring, perhaps at rates three times higher than deeper estuarine waters. Warmer spring waters may be one of the factors influencing biota residing in intertidal regions both in general as well as at our specific sites. We observed greater abundance of fish, and size of *Menidia* sp., in recent (2010–2012) seine surveys compared to similar collections in 1962. While any linkages are speculative and data are preliminary, taken together they suggest that shallow intertidal portions of estuaries may be important places to look for the effects of climate change.

## Introduction

Climate change is a complex and controversial topic and its importance as a driver of ecological research has been increasing, particularly in marine ecosystems. From 1956–2005, global surface (air) temperatures have risen by an average of 0.13°C per decade, which translates to about a 0.65°C rise in temperature over the last ~50 years (1960–2010) [1]. Of course, this is a global average and local temperature trends vary spatially—for example, geographic location and elevation are important factors [2,3]. Also, the methods used to calculate these trends and to account for regional variability, as well as diurnal and seasonal temperature cycles, vary among research groups. This complexity has confused, and often frustrated, both scientists and the public. These challenges are also true for the associated increases in ocean temperatures, particularly along the coasts [1,4]. In estuaries along the east coast of the United States, water temperatures have risen by roughly a degree since the 1960s. Fulweiler et al. [5] observed a 1.4–1.6°C increase in Narragansett Bay, Rhode Island over this period and similar increases have been measured in Woods Hole, Massachusetts [6], Chesapeake Bay [7,8] and the Hudson River Estuary [9]. These values are consistent with the 2014 report from the Intergovernmental Panel on Climate Change which describes an average warming of coastal systems by 0.18±0.16°C per decade over the last 30 years [10].

Warming trends, at least in estuaries, are not consistently observed throughout the year. Daily, monthly, or even seasonal water temperatures have not warmed uniformly over the past 50 years. In Chesapeake Bay, most of the warming has occurred in the winter and spring [7]. This same winter-early spring warming was observed in Woods Hole, on Cape Cod, Massachusetts [6] and Narragansett Bay, Rhode Island [5]. In contrast, long-term data from the Hudson River Estuary indicate the greatest warming occurs later, in the spring and summer [9]. Geographically, the Hudson River Estuary lies between Woods Hole and Narragansett Bay to the north and Chesapeake Bay to the south, so the shifts in seasonal warming cannot be attributed to latitudinal gradients. Seekell and Pace [9] suggest that one reason for this difference, at least between their observations in the Hudson and the Chesapeake, may be that the Hudson is covered in ice in the winter, which probably helps the water to maintain a more constant temperature. This example serves to illustrate the point that understanding, and generalizing about, temperature increases associated with climate change is a challenging task.

A final complication is the potential effect of large and periodic climactic shifts associated with the North Atlantic Oscillation (NAO). In the Northwestern Atlantic, the NAO has been associated with warming periods in the 1930s, 1980s, and 1990s and a cooling period in the 1950s and 1960s. These shifts in temperature, often 1–3°C around long-term means, have affected the ecology of estuarine systems [11]. Locally, in New England, the warmer than average temperatures of the 1930s and 1980s were associated with widespread declines in submerged aquatic vegetation, but a substantial repopulation of the American oyster (*Crassostrea virginica*), as well as a shift from demersal (benthic) to smaller pelagic species [11,12]. The warmer temperatures also shift the timing of phytoplankton blooms, predation by zooplankton, and predation on zooplankton by ctenophores [11]. Warming, in general, seems to speed up the metabolism of the estuary, where warmer water increases growth and productivity, as well as decomposition and respiration. Shifts in the NAO may serve to temporarily exacerbate or mitigate the influence of the more gradual warming of coastal waters associated with global climate change.

Overall, if annual open ocean water temperatures have risen about 0.65°C and east coast U.S. estuaries have increased by about 1.4°C since the 1960s, could temperature increases be even more pronounced in shallow intertidal waters? The challenge in addressing this question is that long-term records of water temperatures from shallow intertidal areas are not readily available. When collecting long-term temperature data, the goal is to achieve the most representative dataset possible. Traditionally, this has meant collecting data from as far away from shore as is feasible to minimize any localized land-based influences like groundwater seeps or stormwater runoff. This is not to say that shallow (<1 m), nearshore water temperature data do not exist, rather that they are not collected in a long-term systematic manner. As it is quick, easy, and inexpensive to take a temperature measurement, water temperature is frequently measured as part of many field studies. But, unfortunately, temperature data are less frequently reported in the peer-reviewed literature. Such data are best found in the gray literature, reports, and the appendices of theses.

**Fig 1:**
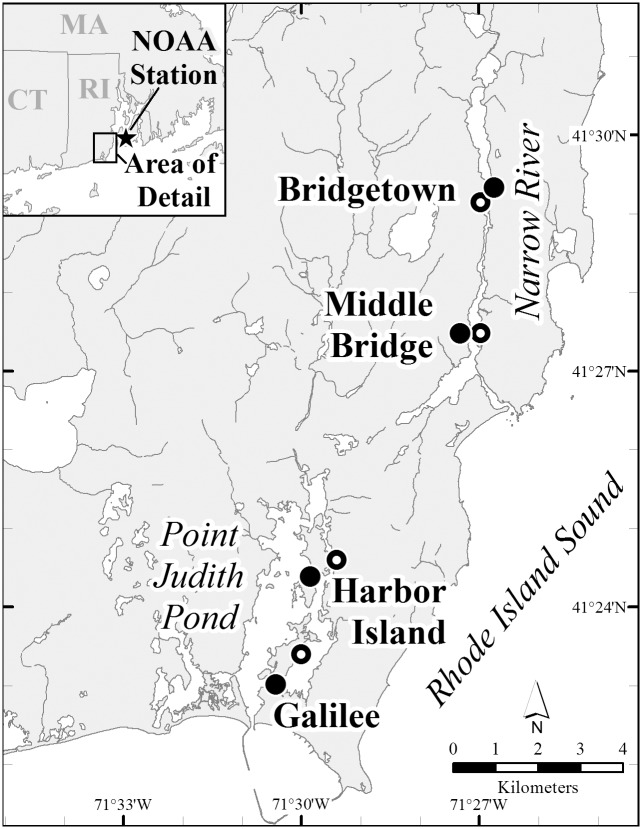
Map of the Narrow River, also called the Pettaquamscutt River, and Point Judith Pond estuary. The four locations sampled by [13] and revisited in 2010–2012 are indicated by closed circles and labeled as Bridgetown, Middle Bridge, Harbor Island, and Galilee. The open circles indicate the location of the deeper water temperature measurements made by [23] and [24]. The location of NOAA’s Newport, RI temperature monitoring station is also noted in the inset map.

We initially set out to repeat a seining study conducted in 1962 [13] with the intent of reassessing intertidal fish populations after 50 years. As part of this effort, we took a closer look at intertidal temperature measurements. While the fish dataset is mentioned, primarily because of the large differences in abundance that we observed, the focus of this manuscript is on the temperature observations. In an effort to assess the factors potentially contributing to the much higher recent fish catch, we compiled all available information on a number of ecological variables (temperature, salinity, nutrient concentrations, population densities, chlorophyll, seagrass extent). From this synthetic effort emerged some tentative, yet potentially profound, observations of shallow intertidal temperature measurements. We have assembled available temperature measurements from 1962–1988 from four sampling locations in Point Judith Salt Pond, a salt water lagoon-type estuary (two locations) and from the Narrow River Estuary, a drowned river valley (two locations), both on Rhode Island’s southern shore (Fig 1). Coupled with these reported measurements are monthly temperature measurements for two consecutive years between 2010 and 2012. The aim of this manuscript is to document some preliminary observations of changes in intertidal water temperature and place these data into an ecological context at each of our four study sites. Intertidal regions, such as these, may potentially be important places to monitor some of the more dramatic effects of global climate change and observe the interactions of warming water temperatures and cultural eutrophication. We hope that this data survey encourages others to look for similar trends in other shallow, intertidal ecosystems.

## Materials and Methods

We coupled data from a master’s thesis [13] which was later presented as a peer reviewed publication [14], with data collected as a part of this study. The comparisons and analyses we present here were limited by the availability of the information presented in these two publications. Additional context, including temperature data, were provided by the available literature and monitoring data. As the four Rhode Island (U.S.A.) stations sampled in the 1962 study have remained accessible, they have been the locations of numerous research projects and theses over the intervening decades. The two stations in the Narrow River are situated in the upper (I, Bridgetown Road at 41°29’14.96”, -71°26’47.90”) and lower (II, Middlebridge at 41°27’28.85”, -71°27’7.44”) estuary which are located 5.63 and 2.25 km, respectively, from the mouth of the estuary (via water, Fig 1). There were also stations in upper (III, Harbor Island at 41°24’24.10”, -71°29’47.77”) and lower (IV, Galilee at 41°22’58.27”, -71°30’25.62”) Point Judith Pond that are 4.33 and 1.63 km, respectively, from the mouth (also via water, Fig 1). No specific permissions were required for these locations and activities as the study sites were accessed through public access points and the regions where we took measurements were below the high-water mark and are thus considered public waters in Rhode Island. This study did not involve endangered or protected species.

### 1962 Study

For his master’s thesis, Mulkana [13,14] conducted a seining study in the summer of 1962 when approximately weekly samples were taken from July 11 to October 20 at two locations in the lower Narrow River and two stations in the Point Judith Salt Pond (Fig 1). Surface water temperature was measured 5 m from shore using a mercury thermometer, which was accurate to ±0.2°C. Surface and bottom salinity were measured using an electronic salinometer (conductivity method) [14]. Seines were cast during an ebb tide using a shore seine (details in [14]) where two seines were conducted for each sampling event (at a particular station and date) and fish abundance data are given as the sum of both seines. Only some fish data are presented in the Mulkana [13,14] publications and the *Menidia* data do not distinguish between the co-existing *Menidia menidia* and *Menidia beryllina*.

### 2010–2012 sample collection

We seined in the same manner as Mulkana [13], at the same locations. While Mulkana sampled approximately weekly from July 11 to October 20, 1962, we sampled monthly beginning on June 22, 2010 and ending on May 23, 2012. In December and January 2010, ice cover prohibited sampling at some stations. Fish captured in the seines were identified and counted. The lengths of the first 30 individuals of each species were also measured for each sampling event. Fish were then released back into the estuary. This sampling was conducted under a RI DEM Division of Fish and Wildlife Scientific Collector’s Permit granted to R. McKinney. To compare the standard length data to our total length data for *Menidia* spp., we used a relationship given in Bengtson [15] where total length = 1.16054×standard length + 1.42.

Both salinity and temperature were measured using a handheld YSI 30 Conductivity, Salinity and Temperature Meter (YSI Incorporated, Yellow Springs, Ohio USA).

### Literature and online data

To provide more context, temperature, nutrients, and productivity data were compiled from the available literature (Table 1). Only measurements made at, or very near, our sampling locations in the shallow, intertidal zone of the estuary (less than ~1 m depth) were included. We also compared our shallow intertidal temperature measurements to those made in adjacent, deeper waters. Monthly temperature data were available from May through October for two deeper water stations in Point Judith via the Salt Ponds Coalition Stations 9650, near our Harbor Island Station (41° 24' 25.20", -71° 29' 31.2"), and 9680, near our Galilee station (41° 23' 16.80", 71° 30' 7.20") [23] for the specific dates (month and year) sampled (Fig 1). Similarly, subtidal water temperature data collected near our Narrow River stations were available during the same time period. These measurements were made by the Department of Environmental Management at their stations NR1 (41° 29' 9.60", -71° 26' 53.30") and NR2 (41° 27' 28.60", -71° 27' 6.90") [24]. We compared the shallow water temperature measurements and corresponding deeper water temperature measurements taken in the same month and year for each of the four stations when deeper water data were available (May-October).

**Table 1:** Data sources and additional details on temperature data used in Figs 2–6. Bridgetown is Station I, Middle Bridge is Station II, Harbor Island is Station III, and Galilee is Station IV. Data locations are shown in a regional context in Fig 1.

Timeframe	Location	Frequency	References
*Intertidal*			
NARROW RIVER			
1962	Bridgetown, Middle Bridge	approximately weekly, July-October	[13,14]
1971, 1973	Bridgetown, Middle Bridge	1–3 times per month, March-May	Graded reports from a zoology course [16,17]
4/1972-3/1973	Bridgetown, Middle Bridge	Approximately twice a month	[18]
2/1976-12/1977	Middle Bridge	twice a month	[19]
11/1978, 12/1978, 4/1979-8/1979, 10/1979	Bridgetown, Middle Bridge	monthly	[20]
6/2010-5/2012	Bridgetown, Middle Bridge	monthly	This Study
POINT JUDITH POND			
1962	Harbor Island, Galilee	approximately weekly, July-October	[13,14]
7/1967-6/1968	Small island west of Harbor Island site, Galilee	twice a month	[21]
2/1976-12/1977	Galilee	twice a month	[19]
4/1980-1/1981	Harbor Island, Galilee	once or twice a month	S. Nixon, unpublished data
5/1987-10/1987 & 5/1988-10/1988	Just East of Harbor Island, Northeast of Galilee	every other week	[22]
5/1990-10/1990	Just East of Harbor Island	every other week	[22]
6/2010-5/2012	Harbor Island, Galilee	monthly	This Study
*Subtidal*			
May to October^a^	Near Bridgetown & Middle Bridge	monthly	[24]
May to October^a^	Near Harbor Island & Galilee	monthly	[23]
1955–1996	Newport	Hourly averaged to Monthly	[11]
1996–2012	Newport	15 minute	[25]

^a^Additional years available, but data from 2010–2012 were used in this study.

Open estuarine water column temperature data available from nearby Newport, RI (41° 30.3’ N, 71° 19.6’ W) were also used. Hourly measurements from 1955–1996 were collected by NOAA and compiled by Oviatt [11]. From 1996–2012, 15 minute data were available online [25]. These data were compiled into mean annual water temperatures, and annual deviations from the mean of the entire dataset were calculated.

### Statistical Analyses

Mean temperature, salinity, and number of total fish per paired seine for the Narrow River and Point Judith Pond collected in the present study were compared with the Mulkana [13] seine study using the Kruskal-Wallis analysis of variance for nonparametric data and the Wilcoxon Rank Sum test was used to examine differences in medians where significant differences were observed. 2010 and 2011 data were combined as the data from both years were not significantly different for temperature, salinity, or total fish catch.

To evaluate the fish length data, we compared 1962 to 2010 and 1962 to 2011 separately. Total lengths were compared per month (July, August, September, October). Mulkana [13] sampled more than once per month, and these data were presented as box and whisker plots per trip (Figures 13–16 in Mulkana [14]), so we used the trip specific data to calculate the traditional ANOVA sums of squares, and combined them to generate an overall monthly standard deviation. We used a traditional two-sample t-test (using the Satterthwaite correction for non-constant variance; the recent data were more variable, most likely due to bimodal distributions) to compare the 1962 data to those from 2010 and 2011. All analyses were performed using SAS 9.3 statistical software. The probability for significance was p<0.05 for all statistical analyses.

## Results and Discussion

### Temperature

**Fig 2:**
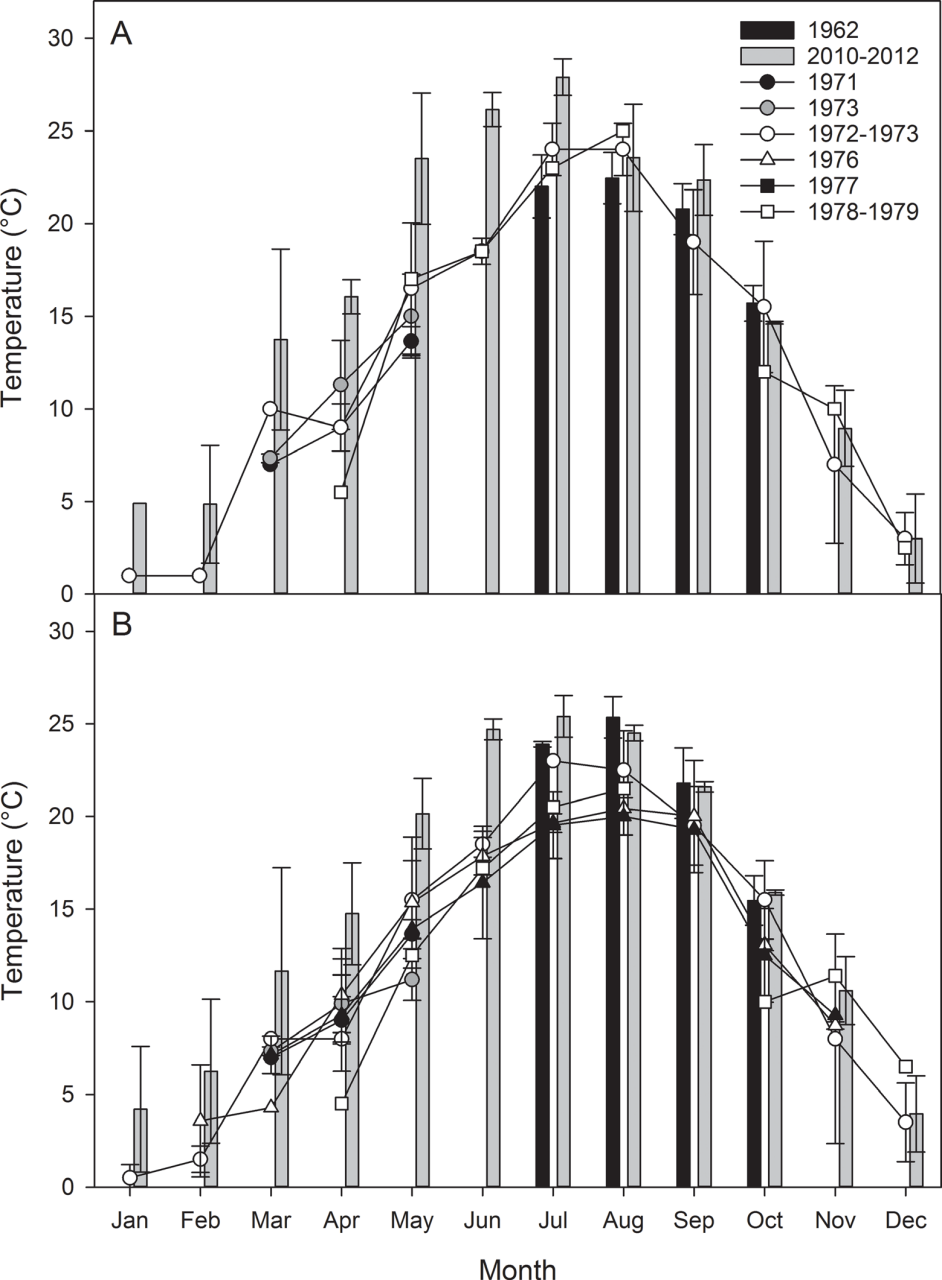
Monthly temperature data for the Narrow River. The top panel (A) presents available monthly temperature data from the shallow intertidal portion of the Upper Narrow River estuary at the Bridgetown Station. The bottom panel (B) presents available data from the Middle Bridge Station. In both panels, black bars represent temperature measured by [13] and gray bars represent temperature measured as a part of this study. Shapes represent data taken from the literature. Bars over data points represent standard deviation. Data sources are listed, and described in more detail, in Table 1.

**Fig 3:**
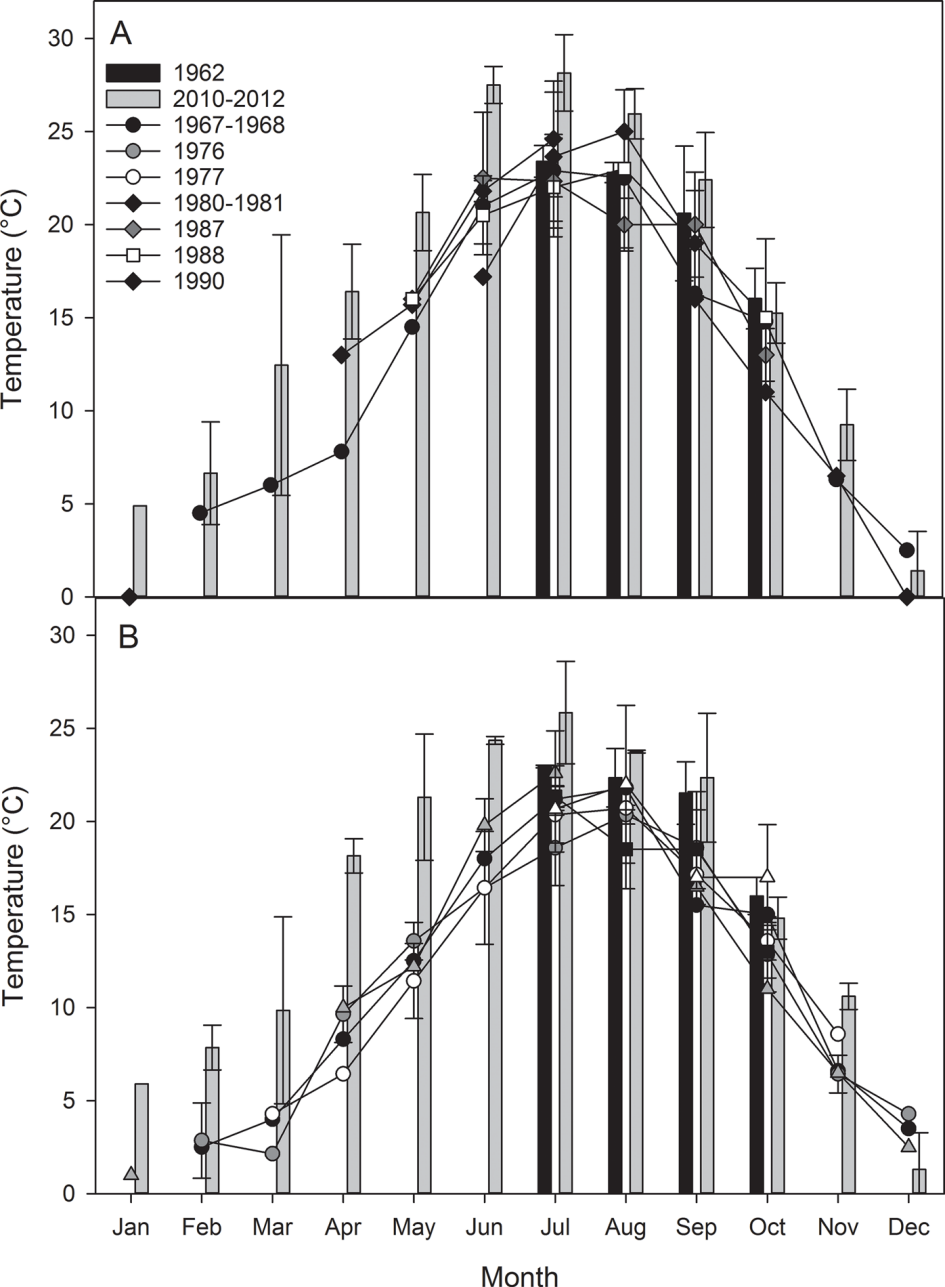
Monthly temperature data for the Harbor Island (upper estuary, A) and Galilee (lower estuary, B) stations in the Point Judith Salt Pond. Black bars represent mean monthly water temperatures measured by [13] and gray bars represent data collected as part of this study. Bars over data points represent standard deviation. Shapes represent data taken from the literature and published reports. For detail on data sources, see Table 1.

**Fig 4:**
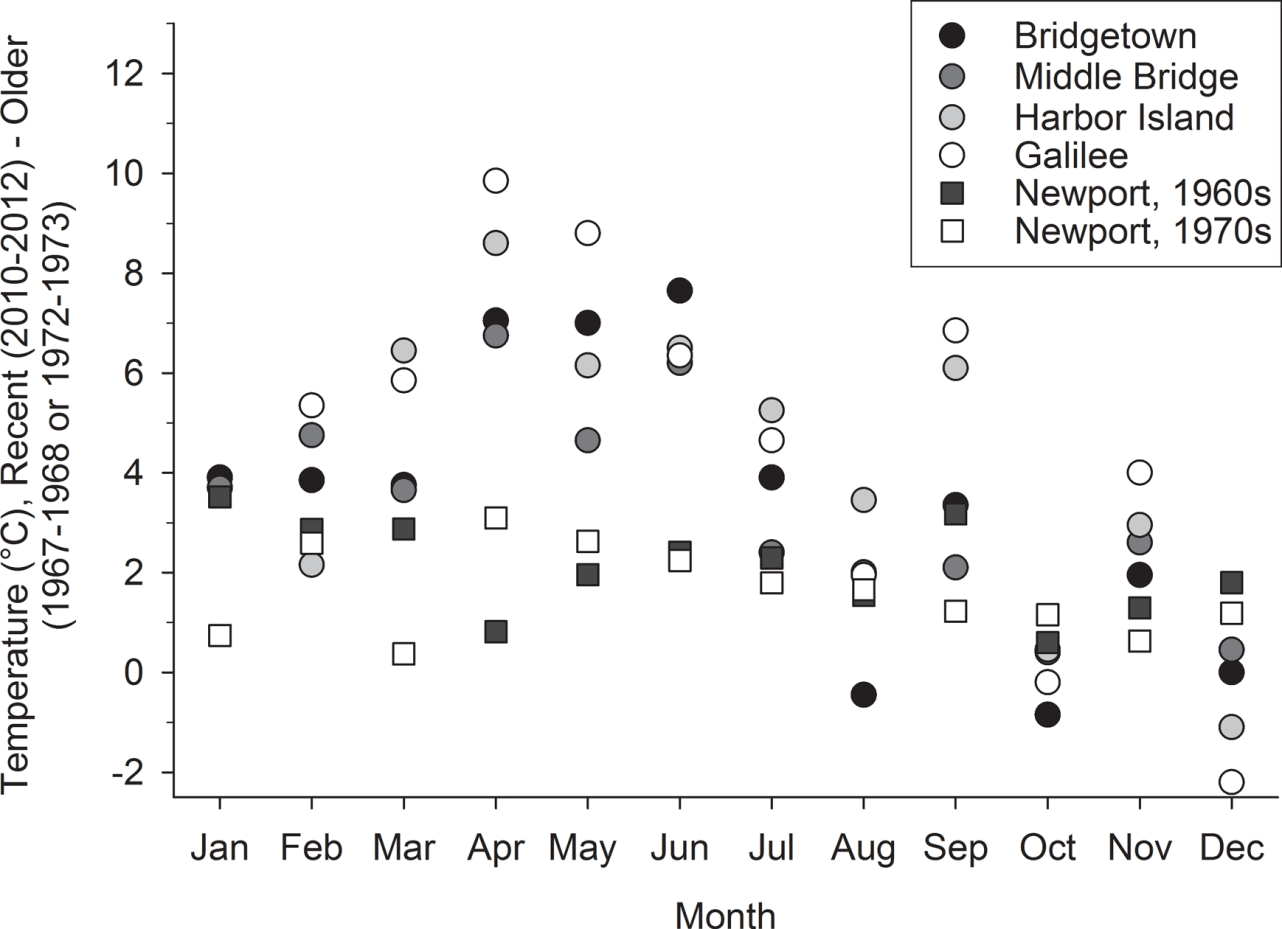
Differences in mean monthly temperatures measured in 2010–2012 (as part of this study) and the earliest full year of available data for our four study locations. The Bridgetown and Middle Bridge (Narrow River) data points represent the difference between the temperature data from this study and measurements made between April 1972-May 1973 [19]. The Harbor Island and Galilee (Point Judith Pond) data points are the difference between this study and data from July 1967-June 1968 [21]. Data from the shallow water stations are shown as open and shaded circles. For reference, we included data from NOAA’s Newport, RI temperature monitoring station [11,25]. The difference between mean monthly temperature measurements in Newport from the time period of this study and both the April 1972-May 1973 and July 1967-June 1968 time periods are shown as closed and open squares. Positive values indicate an increase in temperature over time and negative values indicate cooler temperatures today. The mean of all of the monthly temperature anomalies for all of the shallow water stations, was approximately 3.9°C. If the 2012 data are omitted from the dataset, this value drops to 3.5°C.

Considering both the general seasonal variation in temperature and that associated with climate change in estuaries, a simple, straightforward comparison of summer temperature measurements between 1962 and 2010–2012 might not capture important trends (Figs 2 and 3). Results of t-tests indicated that water temperatures were not significantly different (p>0.05) between Mulkana’s [13] dataset and ours for July through October. However in their study of temperature patterns in Woods Hole Harbor, MA, Nixon et al. [6] observed that most of the increased warming occurred in the winter and early spring months, with little warming evident in the later summer and fall. To evaluate seasonality at our own stations, we analyzed available temperature data from the published and gray literature (Table 1) and the data ranging from 1971–1979 for the Narrow River and from 1967–1990 for Point Judith were used to establish a historical basis for comparison to our 2010–2012 data. The resultant seasonal patterns in the literature data are also similar to those observed by Fulweiler et al. [5] in nearby Narragansett Bay, RI, where the greatest differences appear to be in the winter and spring months. Fulweiler et al. [5] found that annual temperatures increased by about 1.4°C over the past 50 years. The difference between the annual averages of our earliest year-long datasets (1972–1973 for Narrow River and 1967–1968 for Point Judith) and the data we collected (2010–2012) was about 4°C (Fig 4). The differences observed at the four intertidal stations were more dynamic than those observed at the NOAA Newport station, during the same time period [11,25] (Fig 4). When compared to long term trends in water temperature from nearby estuaries [5, 6] the seasonal variations in temperature increase in our shallow water datasets were similar, but the magnitude of the increase was quite different.

**Fig 5:**
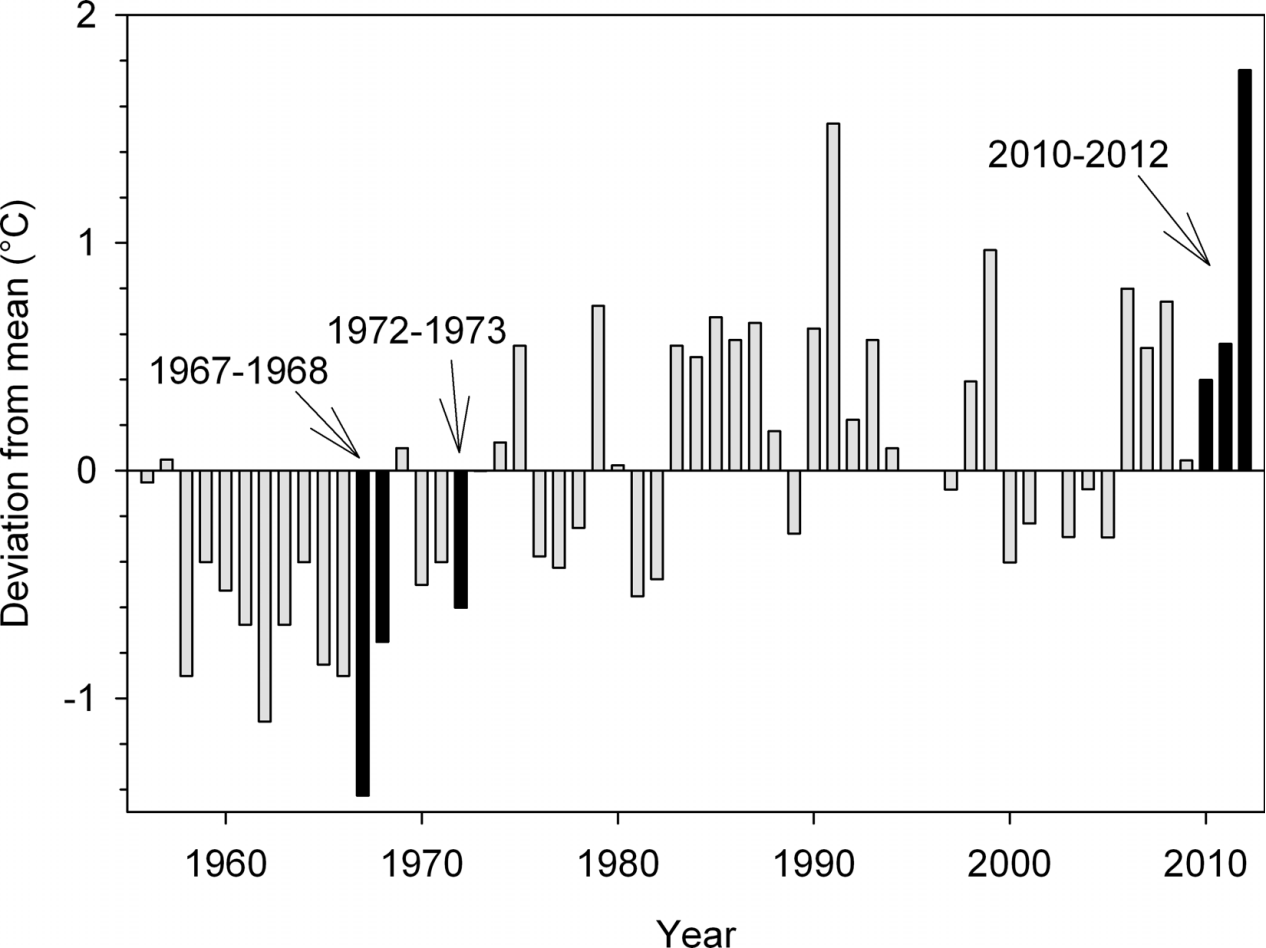
Annual temperature anomalies at Newport, RI (41° 30.3’ N, 71° 19.6’ W). Mean annual temperature data from 1955–2012 were averaged, with the exceptions of 1995, 1996, and 2002 when full years of data were not available (and not shown) [25]. Annual deviations from this average are shown as bars. Positive values indicate mean annual temperatures that are warmer than the long-term average and negative values indicate colder than average annual temperatures. Solid black bars at 1967–1968 and at 1972–1973 are used to indicate the earliest annual data available for Point Judith Pond and Narrow River, respectively, as described in Fig 4. The bar for 1973 is not visible as the annual temperature for that year was so close to the long term average (11.7°C). The years where temperature data were collected as a part of this study (2010–2012) are also indicated by solid bars.

The strong differences in the shallow water data do not appear to be attributable to unusually hot or cold weather during the years when data were available. Annual deviations from the mean of the entire long-term Newport, RI dataset (Fig 5) ranged from about -1 to 1.5°C. While 1967 was the year with the coldest water temperature on Newport’s record, this annual measurement was driven by an unusually cold spring; when the mean of April, May, and June was 9.6°C, compared to means of 11.3°C and 11.5°C for the five years before and after 1967, respectively. This anomalously cold spring likely did not directly influence our temperature dataset at Point Judith, as the first full year of measurements began in July of 1967. Summer temperatures were still cooler than average, but were only about a degree lower than the five years prior to and the five years post 1967. Even if our estuaries also reflected the 1°C cooler summer temperatures observed in Newport, a single degree cannot account for the 6°C or greater increases measured at the Point Judith stations between 1967 and 2010–2012 (Figs 3 and 4). Similarly, 2012 was an unusually warm year for water temperatures in the Northeast Atlantic [26,27] as well as in Narragansett Bay, apparently driven by a winter that was much warmer than the prior two years (Fig 5). Sea surface temperature anomalies off the coast of the Northeastern U.S. were reported to extend until at least June of 2012. There were some other recent temperature data available for Point Judith from 2007–2013, but only for May through October of each year [23]. In looking at the two sampling locations closest to our field sites, temperatures were warmer in May 2012 than in the other years (0.4°C warmer for the northern site and 3.6°C warmer for the southern site). In June and July however, temperature values were neither the warmest, nor among the warmest, values recorded.

When we removed the 2012 temperature data (January-May) from our datasets, the temperature differential (recent minus older data) at the four stations decreased in January, February, and March by ~ 2°C overall (relative to Fig 4). However, the temperature gap in April and May actually widened, and while variable among stations, this increase amounted to about 1°C additional warming. Temperature variations measured at the Newport Buoy in 2012 may not directly translate to our shallow intertidal areas, highlighting the dynamic nature of the data and the challenges associated with generalizing across regions of an estuary. Another good example of regional variability is given by Najjar et al. [8] for the Chesapeake Bay, in which the authors plotted average bay temperatures along with temperature measurements made at the mouths of the York and Patuxent River sub-estuaries. While trends were consistent among the datasets, the magnitude of change was quite different. Temperature differences of 1°C were not unusual between the York and Patuxent estuary mouths [8]. We speculate that differences could reflect local circulation patterns or water column depth where the data were recorded.

Thermal heterogeneity associated with water depth has been documented in streams [28, 29], where temperature differences of up to 7°C have been measured between shallow bank-side and deeper mid-channel waters (e.g. [29]). Shallower waters have a reduced thermal capacity and thus, experience greater fluctuations in temperature [29]. Water depth could also be an important factor in understanding enhanced warming in shallow intertidal estuarine waters. As with rivers and streams, the amount of surface water temperature increase attributable to heat gain from the air is dependent upon water depth, where $\Delta \text{T}=\text{Q}/(\text{H}\times \rho \times {\text{C}}_{\text{p}})$ and ΔT is the temperature increase, Q is heat input, H is water depth, ρ is water density, and C_p_ the specific heat of water [30]. To illustrate the importance of water depth on warming, we can approximate a Q of roughly 100 W m^-2^, where ρ is ~1025 kg m^-3^, and C_p_ is 4200 J kg^-1^°C^-1^ [30,31]. At a depth of 1 m, water temperature could increase by about 2°C d^-1^. In relatively deeper estuarine waters, the temperature increase is much smaller. If mid-estuarine waters were well-mixed and the water depth was 10 m, then the temperature increase would be on the order of 0.2°C d^-1^. While estuarine air-water heat exchange dynamics are certainly more dynamic and complex, with Q values changing with air and water temperature, and processes like mixing and advection also important, this simple exercise illustrates how shallow intertidal areas could be more sensitive to changes in air temperature.

**Fig 6:**
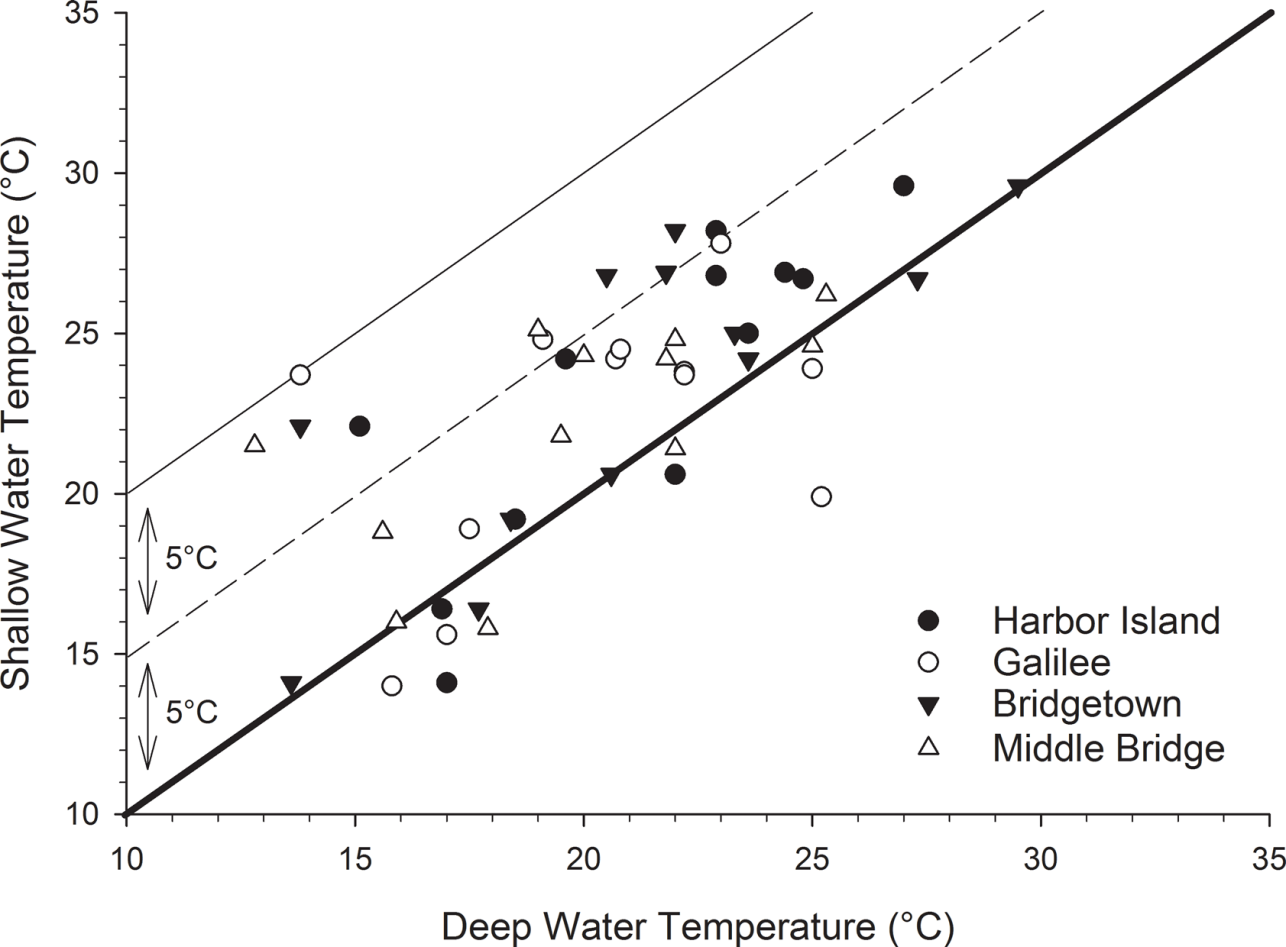
Individual monthly temperature measurements from this study plotted against monthly temperature measurements from nearby deeper waters, collected during the same time period (June 2010-May 2012) [23,24]. Monthly deeper water temperature measurements were available from May to October for each year. The bold black line is the 1:1 line, while the subsequent dashed and thinner solid lines reference the 5°C and then 10°C warmer above the 1:1 line and are included only for reference. Deeper water sample stations are shown in Fig 1.

To further assess the potential importance of water depth on the more recent temperature measurements (June 2010 to May 2012), we compared the shallow water values to available temperature data from nearby deeper water stations (Figs 1 and 6). Both Point Judith Pond and the Narrow River have routine monitoring data available from May to October during our sampling window. The shallow water temperatures are generally warmer than their corresponding deeper water measurements. In contrast to high frequency buoy data, the shallow and deep values were based on single monthly measurements, most likely taken on different days. Given the atmospheric temperature and wind dynamics associated with weather, as well as other factors like tides, currents, and sampling times, we found it remarkable that the shallow water temperatures, across all four stations, were consistently so much warmer than relatively deeper adjacent waters. Tidal ranges are on the order of 44.5 cm in Point Judith and about 13 cm near our Narrow River sampling stations [32,33]. At least for the Narrow River, this translates to a flushing time of about 1.5 days (during average conditions, [32]). While inter-tidal flushing must impact temperatures by mixing the water, in a study of high-frequency temperature measurements from Chesapeake Bay, Preston [7] calculated that daily temperature anomalies were, on average, ±0.2°C from the daily mean. In temperate systems, the amplitude of seasonal temperature variation is much greater, in some cases almost an order of magnitude greater, than tidal and diurnal amplitudes (e.g., [34]). Given the magnitude of temperature changes across seasons compared to those occurring at shorter timescales, our observations of differences between shallow and deeper waters, as well as across time, may not be entirely surprising.

### Salinity

Changes in temperature cannot be entirely attributed to changes in freshwater inflow as there were no differences between salinities measured by Mulkana [13] and the present study at the two lower (more marine) stations and upper Point Judith Pond (p>0.05) (S1 Table). However, salinities at the northern site in the Narrow River (Bridgetown) increased significantly, approximately doubling from about 10–13‰ in 1962 [13] to 18–22‰ in 2010–2012 (χ^2^(1) = 13.76, p = 0.0002). This increase may, in part, be due to improvements made to the bridge crossing the estuary just north of our Middle Bridge site, where the culvert underneath was widened. Though this is a large increase in salinity, it cannot explain the increases in temperature, as temperature rose across all four stations, not just at Bridgetown.

### Other Ecosystem Changes

Although the number of single family residences near our sampling locations has increased greatly since 1962, virtually all of the homes around the Narrow River sites and many of the homes to the east of the Point Judith sites are connected to municipal sewers [35]. Despite this, septic and fertilizer enriched groundwater is estimated to contribute between 28 and 44% of the dissolved inorganic nitrogen to the coastal salt ponds, which includes Point Judith [36]. While nutrient loads have likely increased over the past 50 years, data are not available to quantify the increases at our sites. While nitrate (NO_3_) and phosphate (PO_4_) concentrations were low (<1 μM) in both estuaries in the 1960s (Point Judith) and 1970s (Narrow River) [18,21,37] the absence of ammonium (NH_4_) data makes interpretation difficult. More recent datasets from the Salt Ponds Coalition from 2008–2012 contain both NO_3_ and NH_4_ data and, while NO_3_ values were always <3 μM at both locations, NH_4_ values often exceeded NO_3_, reaching concentrations of up to almost 15 μM. The PO_4_ concentrations were <1 μM in 2008–2012 [23]. Links between nutrient runoff and higher tropic levels, and in particular fish, are complex in New England estuaries [38–40]. For example, abundance and growth rate of *Menidia menidia* did not change with increasing watershed nitrogen loading rates in Waquoit Bay, MA (USA) [39] and there was an inverse relationship between *Fundulus heteroclitus* size and N loads in nearby Narragansett Bay, RI [40].

Although there were no primary production data collected near our Narrow River sampling sites, chlorophyll was measured in Point Judith in both the late 1980s and 2008–2012. Between 1987 and 1990, chlorophyll concentrations were slightly higher near Harbor Island (range 0.4–7.6 μg l^-1^) than near Galilee (0.4–2.8 μg l^-1^), which is expected, as the Harbor Island station in the upper salt pond receives more anthropogenic runoff [41]. More recent seasonal (May-October) average chlorophyll concentrations near Harbor Island ranged from 6.0–8.5 μg l^-1^ in 2008–2011 and up to 19.6 μg l^-1^ in 2012. Near Galilee, concentrations ranged from 1.8 μg l^-1^ in 2008 to 4.9 μg l^-1^ in 2012 [23]. While they qualitatively appear to have increased, chlorophyll concentrations in Point Judith pond are similar to those measured in lower Narragansett Bay, a region considered to be food-limited in the summer months [42–46]. As a recent expansion of eelgrass has been documented near our study sites (from 2009–2012) [47], and nutrient enrichment has been shown to cause a decline in eelgrass (and this effect is exacerbated by warmer water temperatures), we suggest that the eelgrass beds in the Narrow River and Point Judith indicate that water quality is reasonably good in these estuaries [48,49].

**Fig 7:**
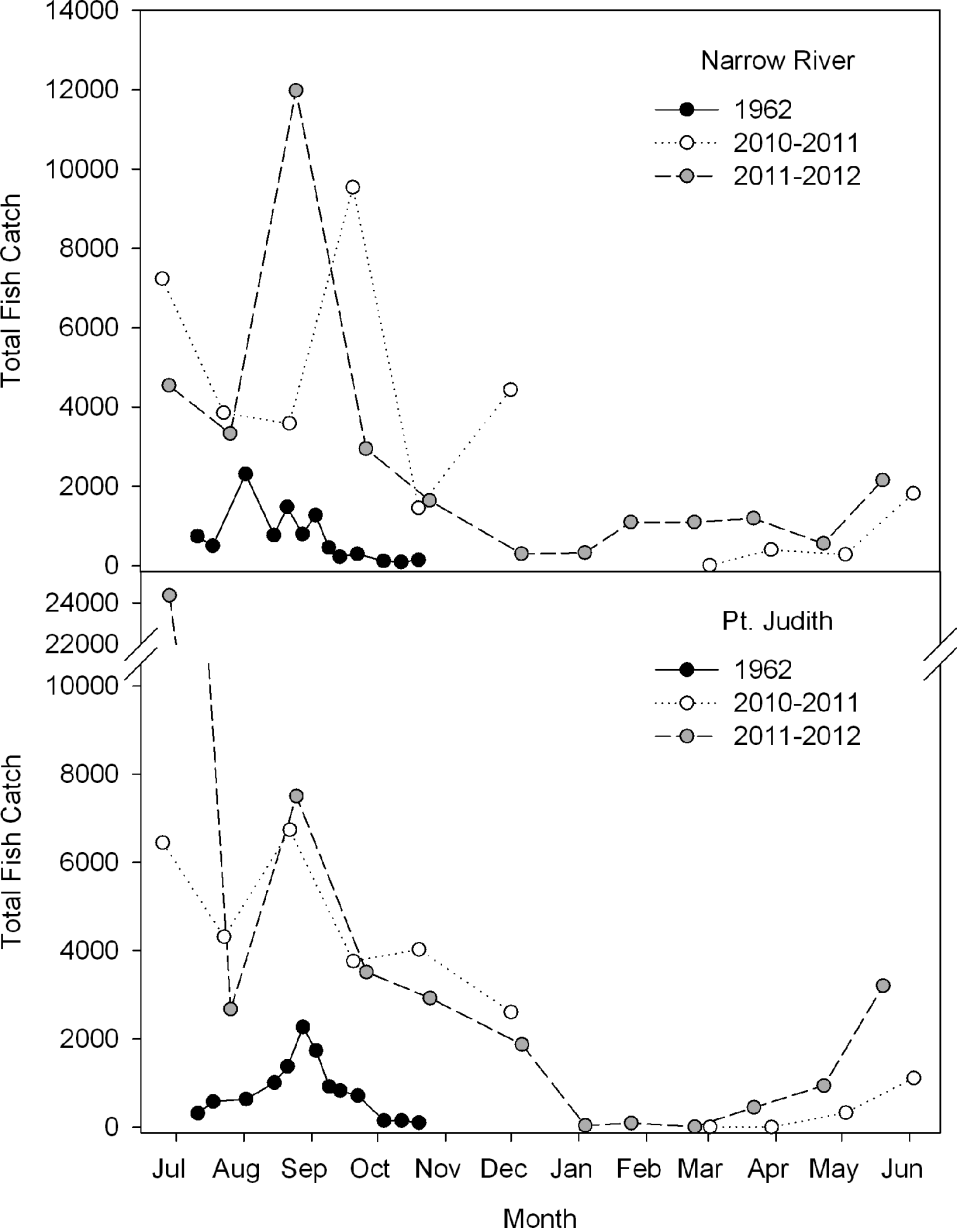
Total catch data from [13] and this study for the Narrow River (Bridgetown + Middle Bridge, top panel) and Point Judith Pond (Harbor Island + Galilee, bottom panel). Mulkana’s data [13] are plotted as closed circles and our data are plotted as open circles (2010–2011) and gray circles (2011–2012). Total catch is the sum of all individual fish captured in each estuary on a given sampling trip. Note the different scales on the Y-axis between the top and bottom panels. As Mulkana began his study in July 1962, the X-axis begins in July and ends in June.

Across stations, our 2010–2012 fish abundance was 4–8 times greater than that measured by Mulkana in 1962 (Narrow River: χ^2^(1) = 12.59, p = 0.0004, Point Judith: χ^2^(1) = 14.19, p = 0.0002, S2 Table). Mulkana [13] began his survey in July and saw a peak in total fish count in August and September with subsequent decline to much lower abundances in October. While we also observed peaks in fish catch during approximately the same time frame, the peaks were much higher, the fall decline later, and then catches dropped close to zero at both estuaries in 2011–2012 (Fig 7). *Menidia* spp. composed between 35% and 52% of the resident species in 1962, their relative abundance increased to 78–87% in the more recent study. The consistency of the two years of recent data suggest that catch values are reasonably representative of current conditions, but the 1962 study provides just one summer of data (July-October) [13], making any comparisons between the 1962 data and the 2010–2012 data notional. We do not know of any other beach seining study conducted in these systems around this time for comparison. However, at least in terms of relative *Menidia* abundance, Mulkana’s data are consistent with other regional seining studies also conducted in the 1960s in Great South Bay, NY and the Slocum River, MA [50,51]. Also, a long-term seining study (1980–2000) from the Hudson River also found an increase in *Menidia* catch over the course of two decades, and the authors cite other locations in New York estuaries where similar observations have been made [52].

*Menidia* length data were available for 1962 and a comparison indicated that those from the 2010–2012 sampling were bigger than those from 1962 in July, August, and September. In these three months, *Menidia* from all four stations were, on average 70% bigger in 2010 and 2011 than in 1962 (p<0.001). In October, fish were not significantly larger at stations I, III, and IV. While we do not have enough data to appropriately assess whether differences between the 1962 and 2010–2012 datasets are real, there is experimental evidence that increasing water temperature exerts a strong positive effect on *Menidia menidia* and, at least, very young *Menidia beryllina* [53,54] and that this is particularly true at higher latitudes [55]. If *Menidia* are growing faster in our intertidal waters, then we would expect them to be bigger in the summer months. The differences we observe suggest that temperature increases could have had an observable, positive influence on fish like *Menidia* over the past five decades—a hypothesis that certainly warrants further investigation.

## Recommendations

**Fig 8:**
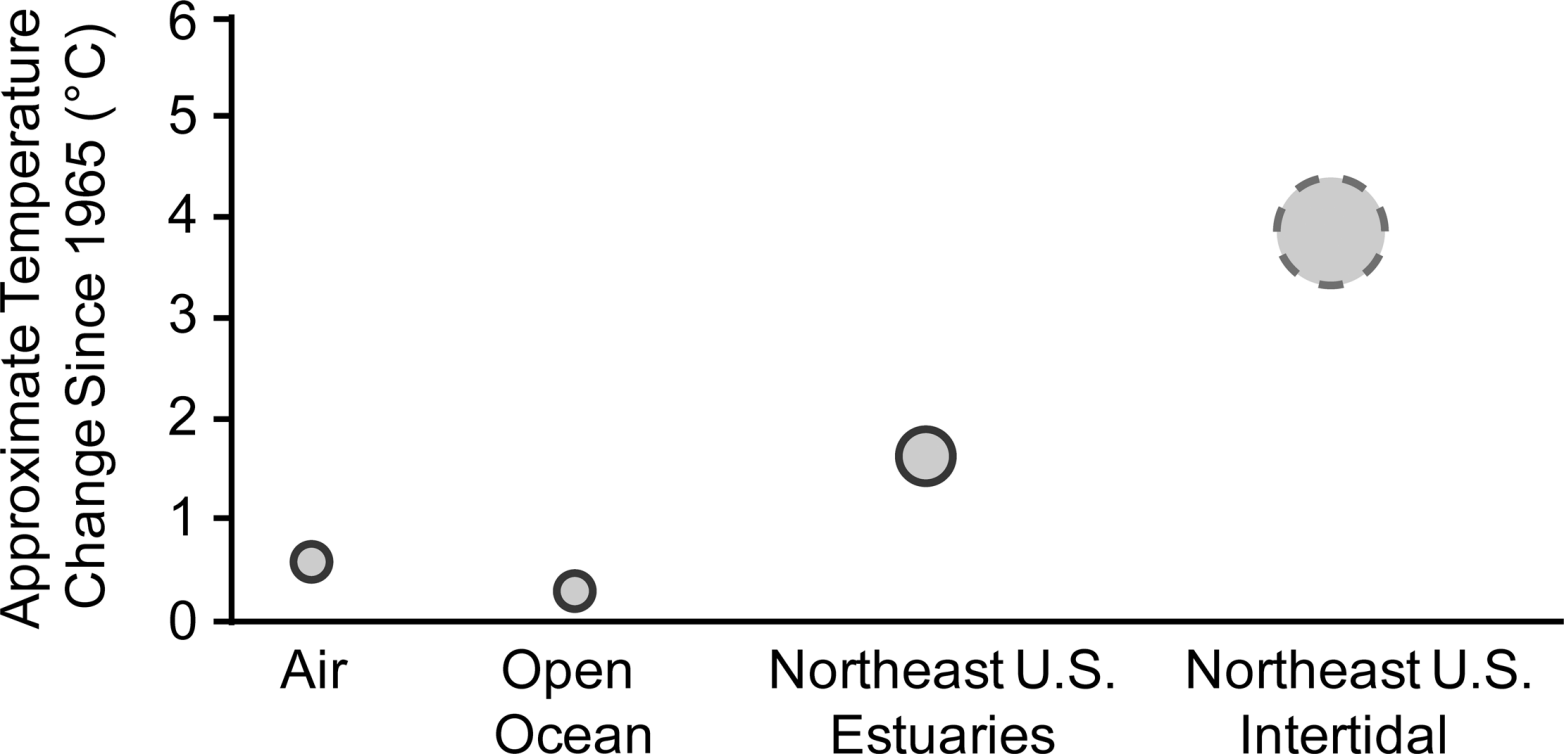
Approximate temperature increases since 1965 are shown for air, the open ocean, Northeastern United States estuaries, and our observations from the shallow intertidal portion of the estuaries. The size of the circle gives a rough approximation of the variability surrounding these measurements. Our observations are denoted with a dashed line as they are less certain. For example, air temperatures have increased by 0.13°C (range 0.10–0.16°C) per decade between 1956 and 2005, or about 0.6°C (range 0.5–0.8°C) since 1965 [1]. Similarly, our estimate of open ocean temperature increase comes from [2] (0.3°C between 1948–1998) in the surface 300 m of the ocean as well as from [56] who showed an approximate rise in sea surface temperature of about 0.4°C since the 1960s. Water temperature data for the Northeast estuaries comes from [5,6], where they observed a temperature increase of ~1.4°C from the 1960’s and 1990’s in Woods Hole, Massachusetts. Data from Boston Harbor, Massachusetts and Newport, Rhode Island are consistent with this trend [6]. The mean annual increase in temperature at our four stations (i.e., Northeast U.S. intertidal) ranged from 3.3–4.7°C, with an average of 3.9°C. If the 2012 data are omitted, this range shifts to 2.5–4.5°C with an average of 3.5°C. The dashed gray circle represents the range of values including 2012.

We observed a substantial rise in intertidal water temperature in the spring, but acknowledge the theoretical error bars are high in this (largely gray literature based) dataset as we have pulled together data from many different studies using different methods and sampled at different periodicities. While we have searched for comparable data from other shallow intertidal waters (<1.5 m deep) elsewhere in southern New England, our investigations have been unsuccessful. We still believe, however, that a collective effort by regional scientists could recover similar datasets. Such efforts would be worthwhile as our preliminary dataset from the Narrow River and Point Judith Salt Pond suggest that the shallow intertidal portions of estuaries may be important overlooked areas to monitor for the potential effects of global climate change. Particularly since intertidal temperatures appear to be increasing more rapidly than in other marine systems (Fig 8).

## Supporting Information

S1 Table Salinity Data for the four study locations in the Narrow River and Point Judith Salt Pond. Station I is Bridgetown, Station II is Middle Bridge, Station III is Harbor Island, and Station IV is Galilee. See Fig 1 for context.
(DOCX)

S2 Table Fish data from 1962 and 2010–2012. Sum of the mean fish abundance of all species and the five most abundant species (based on 1962 data) for resident and migrant marine and brackish water species collected July through October 1962 and 2010–2012 at four stations in the Narrow River and Point Judith Pond estuaries, Rhode Island, USA (Mulkana 1964). Data are reported as the mean number of fish per sampling event (catch per unit effort) where each sampling event was comprised of paired seines. Station I is Bridgetown, Station II is Middle Bridge, Station III is Harbor Island, and Station IV is Galilee.
(DOCX)
