# Supplementary material for: Preliminary Evidence for the Amplification of Global Warming in Shallow, Intertidal Estuarine Waters
Source: PLoS One. 2015 Oct 28;10(10):e0141529. doi: 10.1371/journal.pone.0141529 (PMC4624981; doi:10.1371/journal.pone.0141529)
Supplement: S1 Table — Station I is Bridgetown, Station II is Middle Bridge, Station III is Harbor Island, and Station IV is Galilee. See Fig 1 for context. (DOCX) [file pone.0141529.s001.docx]

**S1 Table. Salinity Data for the four study locations in the Narrow River and Point Judith Salt Pond**. Station I is Bridgetown, Station II is Middle Bridge, Station III is Harbor Island, and Station IV is Galilee. See Figure 1 for context.

|  | 2010-2011 | | | | 2011-2012 | | | |
| --- | --- | --- | --- | --- | --- | --- | --- | --- |
|  | I | II | III | IV | I | II | III | IV |
| June | 12.1 | 30.0 | 29.6 | 29.3 | 17.2 | 24.6 | 29.8 | 31.1 |
| July | 17.3 | 29.5 | 29.6 | 31.1 | 20.1 | 27.8 | 30.2 | 31.3 |
| August | 20.7 | 30.2 | 31.3 | 30.9 | 22.9 | 31.9 | 32.0 | 31.5 |
| September | 21.3 | 26.2 | 26.5 | 28.1 | 22.2 | 28.2 | 29.9 | 31.3 |
| October | 19.7 | 25.7 | 29.2 | 26.7 | 17.2 | 30.8 | 30.2 | 32.0 |
| November | 22.3 | 29.5 | 26.1 | 32.2 | 11.8 | 26.5 | 26.6 | 30.9 |
| December | 17.8 | 24.9 | 16.3 | 24.4 | 15.2 | 26.1 | 24.9 | 24.0 |
| January |  | 26.1 |  |  | 11.2 | 23.4 | 18.2 | 22.3 |
| February | 9.0 |  | 18.5 | 27.9 | 20.4 | 32.1 | 28.7 | 28.4 |
| March | 15.6 | 20.5 | 26.2 | 27.2 | 15.7 | 30.4 | 28.4 | 29.8 |
| April | 15.7 | 29.6 | 28.3 | 30.8 | 18.8 | 31.3 | 26.8 | 30.3 |
| May | 13.6 | 28.3 | 30.9 | 32.8 | 11.2 | 22.2 | 28.1 | 26.4 |
