# Supplementary material for: Preliminary Evidence for the Amplification of Global Warming in Shallow, Intertidal Estuarine Waters
Source: PLoS One. 2015 Oct 28;10(10):e0141529. doi: 10.1371/journal.pone.0141529 (PMC4624981; doi:10.1371/journal.pone.0141529)
Supplement: S2 Table — Sum of the mean fish abundance of all species and the five most abundant species (based on 1962 data) for resident and migrant marine and brackish water species collected July through October 1962 and 2010–2012 at four stations in the Narrow River and Point Judith Pond estuaries, Rhode Island, USA (Mulkana 1964). Data are reported as the mean number of fish per sampling event (catch per unit effort) where each sampling event was comprised of paired seines. Station I is Bridgetown, Station II is Middle Bridge, Station III is Harbor Island, and Station IV is Galilee. (DOCX) [file pone.0141529.s002.docx]

**S2 Table. Fish data from 1962 and 2010-2012.** Sum of the mean fish abundance of all species and the five most abundant species (based on 1962 data) for resident and migrant marine and brackish water species collected July through October 1962 and 2010-2012 at four stations in the Narrow River and Point Judith Pond estuaries, Rhode Island, USA (Mulkana 1964). Data are reported as the mean number of fish per sampling event (catch per unit effort) where each sampling event was comprised of paired seines. Station I is Bridgetown, Station II is Middle Bridge, Station III is Harbor Island, and Station IV is Galilee.
